# Occurrence of Pharmaceuticals in the Seawater Samples of the Port of Cartagena (Murcia, Spain): A Pilot Study

**DOI:** 10.3390/toxics14030217

**Published:** 2026-03-03

**Authors:** Elena Badillo, María Teresa Yuste, Fernando Vallejo, Elisa Escudero, Amnart Poapolathep, Saranya Poapolathep, Pedro Marín

**Affiliations:** 1 Department of Pharmacology, University of Murcia, Espinardo Campus, 30100 Murcia, Spain; 2 Metabolomics Platform, Centro de Edafología y Biología Aplicada del Segura, Consejo Superior de Investigaciones Científicas (CEBAS-CSIC), Espinardo Campus, 30100 Murcia, Spain; fvallejo@cebas.csic.es; 3 Department of Pharmacology, Faculty of Veterinary Medicine, Kasetsart University, Bangkok 10900, Thailand; amnart.p@ku.th (A.P.);

**Keywords:** antibiotics, non-steroidal anti-inflammatory drugs (NSAIDs), pharmaceuticals, potential ecotoxicological risk, port, seawater, SPE

## Abstract

The growing occurrence of emerging contaminants, particularly pharmaceutical residues, in aquatic environments represents a major environmental concern worldwide. While pharmaceutical contamination has been increasingly studied in marine systems, port environments remain largely understudied despite their complex anthropogenic pressures. This study investigates the occurrence, spatial distribution, and potential environmental risk of pharmaceutical residues in surface waters of the port of Cartagena, a multifunctional port on the Spanish Mediterranean coast. Fifteen pharmaceuticals were analysed across nine sampling sites, of which six were not detected. Diclofenac and several antibiotics (erythromycin, azithromycin, clindamycin, and trimethoprim) were the most frequently detected compounds, reaching maximum concentrations of up to 12,294.1 ng/L. Elevated concentrations were observed at sites associated with intense human activity, while the detection of multiple pharmaceuticals at a designated Special Area of Conservation suggests additional diffuse pollution sources, likely linked to insufficient wastewater management in nearby informal settlements. Most detected concentrations exceeded established environmental-quality or risk-threshold values, indicating a potential threat to marine ecosystems. These findings highlight the vulnerability of port environments to pharmaceutical pollution and underscore the need for continuous monitoring programs to support effective environmental management and biodiversity protection in coastal port areas.

## 1. Introduction

The increasing presence of emerging contaminants (ECs), especially pharmaceutical residues, in surface waters has become a significant global concern. This issue has gained growing attention from public administrations and regulatory bodies, leading to a reassessment and modernization of international water quality and environmental protection legislation. Pharmaceutical residues have emerged as ECs of particular interest due to their widespread use, complex physicochemical and biological properties, and persistence in the environment [1,2]. These compounds, commonly used in both human and veterinary medicine, have been detected globally in aquatic environments at very low concentrations, often below the detection limits of routine regulatory monitoring programs. Among these residues, antibiotics, anti-inflammatory drugs, and analgesics are the most frequently detected groups, mainly due to their high consumption rates and potential environmental impacts [3].

In the European Union, the Water Framework Directive (2000/60/EC) provides a comprehensive legal framework aimed at protecting surface waters, groundwater, and coastal waters [4]. The directive’s primary goal is to achieve and maintain good ecological and chemical status in all EU water bodies while promoting continuous improvements in water quality across member states. In line with the EU’s ‘Zero Pollution’ strategy, the legislative framework is being updated to include new substances in the Watch List [5], focusing on freshwater and transitional waters, including estuaries and coastal areas up to one nautical mile from the shore. This list prioritizes emerging contaminants based on their ecotoxicological effects and potential risks to human and animal health.

Although there has been increasing research on pharmaceutical residues in marine environments, port ecosystems have received comparatively little attention. This is alarming given that ports serve as convergence zones for multiple contamination sources, including maritime traffic, urban wastewater discharges, and other human activities. Recent studies have confirmed the presence of pharmaceutical compounds in waters adjacent to ports [6,7], underscoring the urgent need to better understand their behavior and ecological risks in these environments, which are under significant anthropogenic pressure [8]. Current research on pharmaceutical residues in ports primarily focuses on steroids and their metabolites, with studies conducted in fishing ports such as Nagasaki [9], Santos [10], Xiamen [11], Elizabeth [12], and Zierikzee [13]. However, there remains a considerable knowledge gap regarding other pharmaceutical groups, as antimicrobials and non-steroidal anti-inflammatory drugs (NSAIDs), and their ecological implications in port environments.

The Port of Cartagena is a multifunctional port on the Spanish Mediterranean coast that combines commercial, industrial and tourism activities. The port is located in an ecologically sensitive area, overlapping with seven Natura 2000 sites and two Special Protection Areas for Birds (ZEPA), emphasizing its environmental importance. Potential pollution sources in this area include wastewater treatment plants (which often lack effective removal systems for pharmaceuticals) [14], agricultural and urban runoff, maritime transport, and recreational activities like bathing and swimming [15].

Pharmaceutical residues in aquatic ecosystems are a significant concern due to their toxic effects on aquatic organisms such as fish, zooplankton, and benthic species, even at concentrations similar to those found in wastewater effluents. These substances can bioaccumulate in seafood or contaminate drinking water, posing potential long-term risks to human health [16]. Antibiotics, in particular, influence microbial community composition and function by selecting for resistant microorganisms and altering natural microbial populations. This can result in the disappearance or inhibition of certain bacterial groups [17] and affect key ecological processes like biomass production and nutrient cycling [18]. Long-term exposure to antibiotics promotes the development of drug-resistant diseases, a global health challenge that causes approximately 700,000 deaths worldwide. Projections estimate that drug-resistant diseases could cause nearly 10 million deaths per year globally by 2050 [19,20]. Combatting it is a priority in global health agendas. The European Union’s Action Plan on Antimicrobial Resistance and Spain’s National Antibiotic Resistance Plan (PRAN) emphasize the need for environmental monitoring of antibiotics [21,22,23]. The Water Framework Directive supports this effort by facilitating the collection of reliable data on antibiotic concentrations in water, which is crucial for ecotoxicological risk assessments and informed policy-making.

Engaging in the detection and monitoring of pharmaceutical residues is not only progress in terms of near-future water quality standards and the conservation of aquatic ecosystems, but also a key component of a broader health perspective. This approach is in line with the evolving concept of One Health, which addresses emerging challenges at the intersection of environmental, animal, and human health. Considering the current context and the arising needs, this research aims to achieve the following objectives: (i) To determine for the first time in a port area, the presence of pharmaceutical residues and quantify their concentrations in the waters of the Port of Cartagena, (ii) To estimate the existing risk posed by the concentration of drugs detected in Cartagena port’s water.

## 2. Materials and Methods

### 2.1. Chemicals and Reagents

A total of 15 pharmaceutical compounds were analyzed in this study: seven antibiotics (azithromycin, clindamycin, erythromycin, ciprofloxacin, ofloxacin, sulfamethoxazole and trimethoprim), four non-steroidal anti-inflammatory drugs (paracetamol, diclofenac, ibuprofen and naproxen), two antifungals (clotrimazole and fluconazole), one antidepressant (venlafaxine) and one oral antidiabetic drug (metformin). The selection of the antimicrobials, which are to be analyzed, is based on List of observations published by the EU, the reports of the Spanish Agency for Medicines and Health Products (AEMPS) and the European Medicines Agency (EMA) about the consumption of antibiotics in Spain and Europe, as well as on previous data on their occurrence in seawaters and wastewaters analyzed in Mar Menor and other parts of Spain [24,25,26]. Β-lactams have not been included due to these antibiotics are highly unstable and subjected to polymerization, molecular rearrangement and reactions encompassing hydrolysis. As a result, compounds from this class of antibiotics are infrequently found in environmental waters [27].

All pharmaceuticals were purchased from Cymit Química (Barcelona, Spain). Stock solutions (1 g/L) were prepared by dissolving the appropriate amount of pharmaceutical standard in either 100% methanol or HPLC-grade water (Merck Life Science, Madrid, Spain), depending on the solubility of the compound. Working solution mixtures for analysis and calibration were prepared by serial dilution in methanol or water. All solutions were stored at −40 °C.

### 2.2. Sample Collection

**Figure 1:**
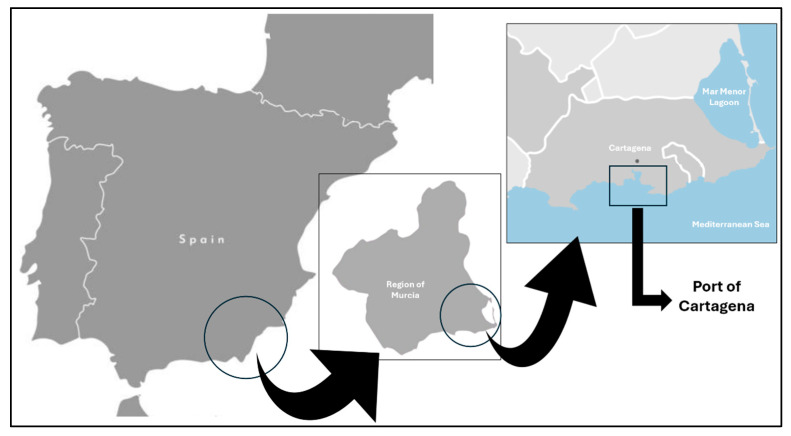
Location of the Port of Cartagena (Murcia, Spain).

The Port of Cartagena is located in the southeastern region of Spain, on the European Mediterranean coast, playing a key role in maritime connectivity between Europe, Africa, and the Mediterranean (Figure 1).

**Figure 2:**
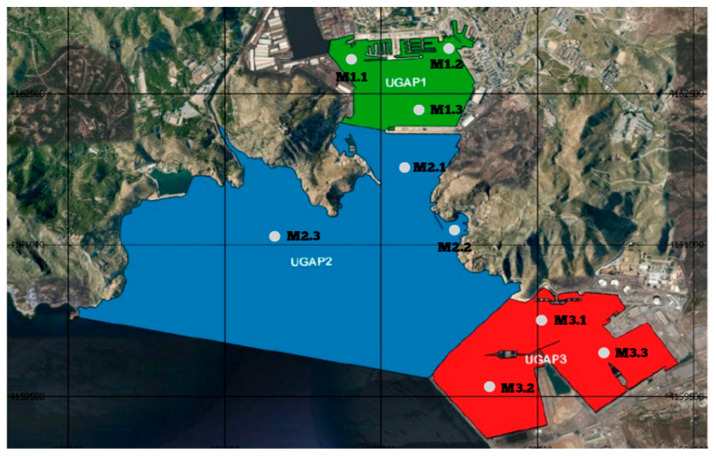
Sampling stations for water collection in the Port of Cartagena. Image obtained from UGAP. The Port Water Management Unit is defined based on its uses, geomorphology, discharge history and water body type. Source: APCC.

The nine sampling stations in the Port of Cartagena (Figure 2) were selected based on the three defined water bodies: Cartagena Dock (Water Body 1), Escombreras Dock (Water Body 2) and the areas outside the docks (Water Body 3). The selection criteria included the areas with the highest activity pressure, as well as existing sampling points established for port water quality monitoring.

- Marina (M1.1)
- Fishing Dock (Santa Lucía Pier) (M1.2)
- Cruise Terminal ‘Juan Sebastián Elcano’ (M1.3)
- Cruise Entry Zone (M2.1)
- Cala Cortina (M2.2).
- Sampling point in the Special Conservation Zone (ZES) ES6200048 (M2.3)
- Escombreras Dock Point 1 (M3.1)
- Escombreras Dock Point 2 (M3.2)
- Escombreras Dock Point 3 (M3.3).

The sampling campaign was conducted in February 2025. Samples (approximately 2 L) were collected from the surface (at a depth of less than 0.5 m) at the previously described sampling points. At each site, one independent water sample was collected. The samples were stored in 2.5 L amber glass bottles that had been pre-rinsed with methanol and deionized water. The samples were kept in the dark under refrigeration and analyzed within 48 h of collection. Each sample was analyzed in triplicate for the determination of pharmaceutical concentrations.

During the sampling campaign, the following physicochemical parameters were also measured in situ at different stations: temperature, electrical conductivity, pH, dissolved oxygen, salinity, chlorophyll concentration and oxidation-reduction potential (Table S1).

### 2.3. Chromatographic Conditions

Analysis of emerging contaminants was performed using a 1290 Infinity II UHPLC system (Agilent Technologies, Waldbronn, Germany) coupled to a Triple Quad™ 6500+ mass spectrometer (Sciex, Wilmington, DE, USA). Separation was achieved using a Poroshell 120 Bonus-RP C18 column (3.0 × 100 mm, 2.7 µm, Agilent Technologies, Waldbronn, Germany). A sample volume of 2 μL was injected at a flow rate of 0.4 mL/min. The mobile phases consisted of 0.1% formic acid in LC-MS grade water (solvent A) and acetonitrile (solvent B).

Chromatographic separation was performed using the following gradient conditions: 0–2 min: 1–20% solvent B; 2–4 min: 20–40% solvent B; 4–6 min: 40–60% solvent B; 6–8 min: 60–99% solvent B, held for two minutes. The system then returned to the initial conditions (1% B) over two minutes, and the column was re-equilibrated for an additional two minutes, resulting in a total analysis time of 14 min.

The turbo ion spray interface was used in the electrospray ionization mode with polarity switching. The ion source parameters were as follows: curtain gas at 35 psi, CAD gas at 9 psi, ion spray voltage at 5500 V/4500 V, temperature at 400 °C, gas 1 at 60 psi and gas 2 at 60 psi, being both of them pure nitrogen (99.999%). The total acquisition time was 14 min. Analysis was performed in multiple reaction monitoring (MRM) mode using the scheduled MRM™ algorithm with a 60-s time window. Two ion transitions were monitored per compound: one for quantification (MRM1) and one for confirmation (MRM2). Data acquisition and quantification were carried out using Sciex OS 2.2.0.5738 software (Sciex, Wilmington, DE, USA. Tables S2 and S3 present the mass spectrometry parameters used to determine the target analytes in negative and positive ionization modes, respectively (Tables S2 and S3). Representative MRM chromatograms corresponding to the standard sample, spiked sample, and environmental sample are provided in Figures S1–S3, respectively.

### 2.4. Solid Phase Extraction

Solid-phase extraction (SPE) of analytes was carried out using Strata-X cartridges (33 µm, 200 mg/6 mL, polymeric reversed-phase; Phenomenex, Madrid, Spain), based on the method previously described [28]. Prior to extraction, 250 mL of each water sample was filtered through a 1.2 μm glass fibre filter. No pH adjustment was applied prior to SPE because satisfactory recoveries were obtained in the native seawater matrix. Cartridges were conditioned with 6 mL of methanol followed by 6 mL of HPLC-grade water, both applied under vacuum (400 mbar). After sample loading, cartridges were washed with 6 mL of HPLC-grade water and dried under vacuum for 15 min. Elution was performed with 6 mL of methanol and 3 mL of a methanol:dichloromethane (50:50, *v*/*v*) mixture. Potential clogging of SPE cartridges due to the complex port water matrix was effectively avoided by the pre-filtration step and by loading samples at a controlled flow rate. The eluates were evaporated to dryness under a gentle stream of nitrogen at 40 °C and reconstituted in 1 mL of methanol.

### 2.5. Quality Control

#### 2.5.1. Calibration and Quantification

Calibration curves were constructed individually for each target analyte using at least ten concentration levels prepared by serial dilution of stock solutions in methanol or water, according to analyte solubility. The calibration range for each compound was defined starting at the validated limit of Quantification LOQ and extending to concentrations exceeding the highest levels detected in environmental samples (Table 1). Linear regression models were applied for all analytes, yielding coefficients of determination (R^2^) ≥ 0.995. Quantification was performed by external calibration using the peak area of the quantifier transition (MRM1) acquired in scheduled MRM mode. Data acquisition and processing were carried out using Sciex OS software (v2.2.0.5738). To compensate for matrix-induced ion suppression or enhancement associated with seawater salinity, matrix-matched calibration curves were applied.

#### 2.5.2. Limits of Detection and Quantification

The limits of detection (LOD) and limits of quantification (LOQ) were determined during method validation based on signal-to-noise (S/N) criteria. The LOD was defined as the lowest concentration producing a signal-to-noise ratio ≥ 3, while the LOQ corresponded to the lowest concentration producing a signal-to-noise ratio ≥ 10, together with acceptable precision and recovery. The reported values correspond to method detection limits, including solid-phase extraction (SPE) recovery and matrix effects, rather than instrumental detection limits. The analytical method showed a wide range of sensitivity depending (Table 1). Limits of detection (LOD) ranged from 10 ng/L (e.g., clindamycin, erythromycin, sulfamethoxazole, trimethoprim and diclofenac) to 5000 ng/L for compounds such as ciprofloxacin and ibuprofen. Correspondingly, limits of quantification (LOQ) varied between 25 ng/L and 10,000 ng/L. Higher LOQ values were mainly observed for certain fluoroquinolones, NSAIDs, and highly polar compounds (e.g., metformin). For analytes reported as non-detected (ND), concentrations were treated as excluded from RQ calculations in subsequent analyses. For compounds with comparatively high LOQs (e.g., ciprofloxacin and ibuprofen), non-detections should be interpreted with caution, as concentrations below the LOQ cannot be excluded. This methodological constraint may result in an underestimation of calculated risk quotients (RQs), since actual environmental concentrations could be present at levels below the quantification limit.

#### 2.5.3. Recovery

Method recovery was evaluated by spiking seawater samples with known concentrations of each analyte prior to SPE extraction. Spiked samples were processed following the same procedure as environmental samples, including filtration, extraction, evaporation, and reconstitution. Recoveries were calculated by comparing measured concentrations in spiked seawater extracts with those obtained from calibration standards. Compound-specific recoveries ranged from 70% to 114% (Table 1), which are considered acceptable for complex marine matrices. Slight signal enhancement was observed for clindamycin (114%), likely attributable to matrix effects. Higher LOQs observed for ciprofloxacin, ibuprofen and naproxen were mainly attributed to stronger ion suppression during electrospray ionization combined with moderate SPE recoveries (approximately 70%), reflecting the challenges associated with seawater analysis.

#### 2.5.4. Measurement Uncertainty

Measurement uncertainty was estimated from intra-day precision and recovery variability. Expanded uncertainty (k = 2) was below 30% for all analytes, which is considered acceptable for trace analysis in complex environmental matrices.

### 2.6. Risk Assessment

The risk assessment was conducted based on studies documenting the potential effects of selected pharmaceuticals on invertebrates, mollusks and fish. The Risk Quotient (RQ) was calculated by comparing the Measured Environmental Concentration (MEC) with the Predicted No-Effect Concentration (PNEC), with the MEC/PNEC ratio indicating actual ecological risk. Risk levels were categorized as follows: RQ > 1 indicates high risk, 0.1 ≤ RQ < 1 indicates moderate risk, 0.01 ≤ RQ < 0.1 indicates low risk, and RQ ≤ 0.01 indicates insignificant risk [29].

RQ = MEC/PNEC

The PNEC values used are based on those established in the review studies [30], which were in turn based on data from various species of invertebrates, mollusks and fish. Table S4 shows for each detected compound the PNEC value used, the test organism, the toxicity endpoint (LC50), and the assessment factor applied (AF) [31,32,33,34,35,36,37,38,39,40]. When multiple ecotoxicological endpoints were available for a compound, the lowest reliable value was selected for PNEC derivation following a precautionary approach, in order to avoid underestimation of environmental risk. Acute LC50 or EC50 values were divided by an assessment factor (AF) of 1000, whereas chronic EC10 values were divided by an AF of 50, in accordance with standard environmental risk assessment practices.

In the case of antibiotics, the PNEC-MIC (minimum inhibitory concentration) approach was also considered [41]. This metric represents a concentration threshold below which the selection of antibiotic-resistant bacteria in the environment is not expected to occur. 

PNEC values aimed at preventing the selection of antibiotic resistance (PNEC-MIC) were adopted from Bengtsson-Palme and Larsson (2016) [41]. To determine this value, the lowest reliable MIC available for the antibiotic in question is identified, and a safety factor of 10 is applied to ensure that the allowable environmental concentration remains well below the threshold that could exert selective pressure on bacterial populations. The microbial risk quotient (RQ_MICsub_) was evaluated based on the predicted minimum concentrations required to select for antibiotic resistance, taking into account each antibiotic’s presence in surface water.

The microbial risk quotient (RQ_MICsub_) was calculated as:

RQ_MICsub_ = MEC/PNEC_MIC_

where MEC represents the measured environmental concentration in surface water.

Values of RQ_MICsub_ > 1 indicate a potential risk for selection of antibiotic resistance in the aquatic environment.

Given the pilot nature of this study and the single sampling campaign design, the calculated RQ and RQ_MICsub_ values should be interpreted as screening-level estimates under the specific environmental conditions at the time of sampling.

### 2.7. Statistical Analysis

Descriptive statistics, including means, ranges, and minimum and maximum values, were calculated using Microsoft^®^ Excel^®^ for Microsoft 365 MSO (version 2505, build 16.0.18827.20102, 64-bit; Microsoft Corporation, Redmond, WA, USA). Additionally, a detection frequency graph representing the percentage of detection per compound was generated using the same software for data visualization.

## 3. Results

Six of the fifteen pharmaceuticals analyzed (clotrimazole, ibuprofen, naproxen, paracetamol, venlafaxine and metformin) were not detected at any of the nine sampling sites. However, non-detections of compounds with higher LOQs should be interpreted with caution, when calculating risk, as concentrations below the LOQ cannot be ruled out.

In Table 2, the mean, minimum and maximum concentrations of the detected pharmaceuticals at the various sampling locations are presented, as well as their detection frequencies.

**Figure 3:**
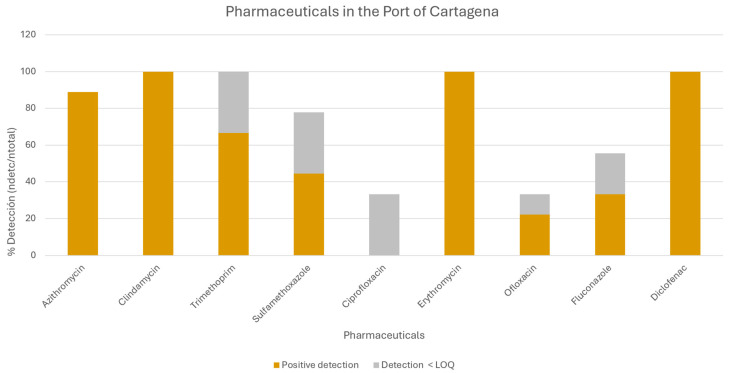
Detection frequency of target compounds expressed as the percentage of positive detections across all analyzed samples.

The detection frequency of each target compound was evaluated and expressed as the percentage of positive detections across all samples (Figure 3). This analysis allowed identification of the most commonly detected compounds.

The most frequently detected pharmaceuticals were the anti-inflammatory drug diclofenac and the antibiotics erythromycin, azithromycin, clindamycin, and trimethoprim. The maximum concentrations of these active substances in seawater were 5880.9; 435.5; 2206.5; 608.65 and 128.71 ng/L, respectively. Table 3 shows the average concentration of the drugs detected at each sampling point (ng/L).

The sampling points corresponding to Marina (M1.1), Cala Cortina (M2.2) and ZEC ES6200048 (M2.3) had the highest accumulation of the detected pharmaceuticals. Specifically, azithromycin, erythromycin, fluconazole and trimethoprim were most concentrated at the marina, while diclofenac and sulfamethoxazole were most concentrated at Cala Cortina.

**Figure 4:**
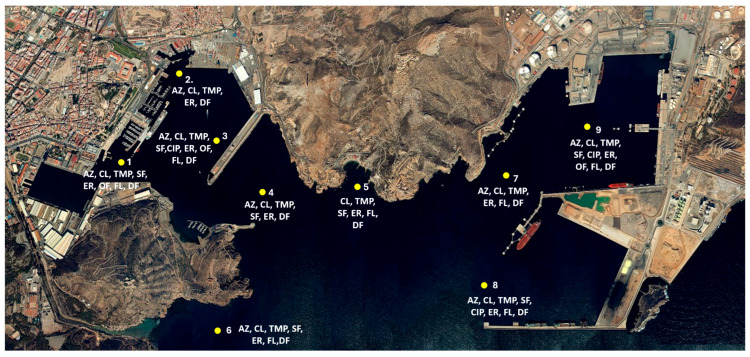
Pharmaceuticals detected at each sampling point in the Port of Cartagena. Azithromycin (AZ), Clindamycin (CL), Trimethoprim (TMP), Sulfamethoxazole (SF), Ciprofloxacin (CIP), Erythromycin (ER), Ofloxacin (OF), Fluconazole (FL), Clotrimazole (CT), Diclofenac (DF), Ibuprofen (IB), Naproxen (NA), Paracetamol (PT), Venlafaxine (VF), and Metformin (MT).

The antibiotics azithromycin, clindamycin, trimethoprim, sulfamethoxazole and erythromycin, along with the anti-inflammatory diclofenac, were detected at the control site (ZEC ES6200048 sampling point). One possible explanation for the presence of these compounds at this supposedly low-impact site is its proximity to the informal settlement of Algameca Chica, which is located at the mouth of the Benipila watercourse. This area drains directly into the sea and lacks proper sewage and wastewater treatment infrastructure. Figure 4 illustrates the presence of various pharmaceuticals at each sampling location and how they are distributed throughout the Port of Cartagena. The greatest diversity of pharmaceutical compounds was observed in the Cartagena Dock (points 1 and 3) and the Escombreras Dock (points 8 and 9).

## 4. Discussion

The Water Framework Directive (WFD 2000/60/EC) is the European Union’s main regulatory framework for protecting and improving water quality [4]. It establishes a list of priority substances that must be monitored and whose composition is periodically reviewed (Article 16.4). With the entry into force of Directive 2013/39/EU, Article 8b was introduced, enabling the European Commission to establish a watch list aimed at collecting data on substances with potential environmental risks that have not yet been classified as priority substances [42]. In 2013, diclofenac (DF) and two estrogenic hormones were included in the initial watch list, marking a milestone in the regulatory focus on pharmaceuticals in the environment. Subsequently, Decision 495/2015 (EU Commission 2015) expanded the list to include ten compounds, adding three macrolide antibiotics (clarithromycin, azithromycin and erythromycin) recognized for their widespread use and frequent detection in wastewater and surface waters [5]. Regulatory interest in these pharmaceutical compounds (such as diclofenac and macrolide antibiotics) arises from their persistence, their potential to bioaccumulate, and their ecotoxicological effects. Including such contaminants in regular monitoring programs is a proactive approach that anticipates their possible future classification as priority substances in upcoming WFD updates.

Diclofenac is a non-steroidal anti-inflammatory drug (NSAID) that is widely used in both human and veterinary medicine. Since the 2000s, multiple studies have identified it as one of the most frequently detected pharmaceuticals in the environment. Concentrations in coastal seawater have been reported to vary widely. Elevated levels of diclofenac were detected in open marine waters along the Red Sea coast in Saudi Arabia, reaching up to 10,200 ng/L [43]. Several studies suggest that the continuous release of partially treated wastewater into the ocean, combined with limited biodegradation capacity, leads to significant accumulation of this pharmaceutical in the marine environment [44,45]. Likewise, substantial diclofenac concentrations have been reported in other regions: 1500 ng/L in the Mediterranean Sea [46]; 550 ng/L off the west coast of Ireland [47]; and 241 ng/L in the Atlantic Ocean [48]. These findings are associated with increased maritime activity and higher visitor numbers in these areas.

The maximum concentration of diclofenac measured in the port area of Cartagena falls within this range, at 5882.98 ng/L. This is relatively high compared to previously reported data. In this case, the specific characteristics of the port must be considered, such as reduced water volume, limited surface water movement and anthropogenic pressure, as well as potential entry points mainly linked to the discharge of treated wastewater and human activities.

Reports on diclofenac concentrations in open marine waters are more limited. In such environments, concentration ranges tend to be lower (0.02–16.3 ng/L) due to sorption, degradation and dilution processes [49]. Diclofenac was detected in coastal areas at relatively low concentrations. A study conducted across 43 coastal and offshore sites in the Baltic Sea reported diclofenac concentrations ranging from 2 to 51 ng/L, with a median value of 26 ng/L [50]. Compared to the current study, the concentrations reported in these areas are notably lower.

The environmental presence of azole antifungals has become an emerging concern, not only due to their ecotoxicological impact but also because of their potential role in promoting the development of drug-resistant fungi [51]. One notable example is fluconazole (FL), a widely used azole fungicide commonly found in oral and topical medications for treating fungal infections [52]. However, FL is also present in various household products such as soaps, shampoos, dermal creams and shower gels [53], increasing the likelihood of its release into the environment and contributing to its widespread distribution and associated ecological risks. This antifungal is characterized by its high persistence and mobility in aquatic environments. It has been widely detected in surface waters worldwide. Due to its limited removal efficiency during conventional wastewater treatment processes, FL is likely to persist in the environment, posing potential risks to both aquatic ecosystems and human health. Furthermore, its degradation products, such as 1,2,4-triazole and 1,2,4-triazole-1-acetic acid, exhibit even greater persistence, with half-lives ranging from 1 to 3 years [51].

Fluconazole has been detected in a previous study in open seawater at concentrations ranging from approximately 0.5 to 25 ng/L, with an estimated mean concentration around 6 ng/L in samples collected from coastal and offshore areas of the Baltic Sea [50] and from 0.51 to 22 ng/L (medium: 0.69 ng/L) in the coastal water of South Pacific [54]. In the current study, however, the mean concentrations were significantly higher, with average values of 428.55 ng/L and maximum levels reaching 718.31 ng/L. Regional differences such as higher local consumption, proximity to wastewater discharges, and environmental conditions that reduce degradation rates may contribute to the elevated concentrations observed in our samples.

The presence of antimicrobials is closely linked to the significant issue of antimicrobial resistance, one of the greatest challenges to global public health [55]. Azithromycin is a semi-synthetic macrolide antibiotic derived from erythromycin. It belongs to the macrolide class and is widely used to treat infections in humans and animals. Azithromycin is the most prescribed antibiotic for respiratory tract infections, and its use has increased substantially since the start of the SARS-CoV-2 pandemic [56]. Various studies have documented different concentrations of azithromycin in marine environments, reflecting spatial and temporal variability. In China, mean concentrations of 14 ng/L were detected in mariculture areas [57], whereas coastal waters exhibited lower concentrations, averaging 5 ng/L [58]. During the SARS-CoV-2 pandemic, azithromycin concentrations of approximately 7 ng/L were reported in both the winter and summer near the Port of Bushehr, Iran, indicating persistent usage of the antibiotic during this period [59].

By contrast, the present study, conducted in the Port of Cartagena (Murcia, Spain), revealed substantially higher mean azithromycin concentrations (838.28 ng/L, ranging from 232.2 to 2206.7 ng/L) alongside higher erythromycin concentrations (172.95 ng/L, ranging from 64.6 to 445.5 ng/L). These values exceed the maximum concentrations reported in other marine locations, such as the Mar Menor (40.7 ng/L for erythromycin and 154.7 ng/L for azithromycin) [26], and various coastal waters in China [60]. Higher erythromycin levels have only been documented in the South China Sea, where concentrations reach 1900 ng/L [61]. Taken together, these findings suggest that the environmental burden of macrolide antibiotics is significantly higher in the Port of Cartagena region than in other studied coastal areas.

In the case of clindamycin, no studies to date have quantified its concentrations in seawater. However, its persistence in the environment is supported by evidence of its poor removal efficiency in wastewater treatment plants [61]. This is illustrated, for instance, by a study on the occurrence of emerging contaminants in the urban water cycle in Latin America, where clindamycin was detected in water samples from Colombia and Costa Rica. Mean concentrations were reported at 2158.75 ng/L in wastewater, and at 8.0, 20.0, 11.0, and 13.0 ng/L in surface water, groundwater, effluent, and influent samples, respectively [62].

Fluoroquinolones are a class of widely used antibiotics in marine aquaculture, resulting in their frequent detection in aquatic environments. Ofloxacin, one of the most used fluoroquinolones, is particularly prevalent in aquaculture waters and adjacent coastal areas due to its extensive application [63]. Concentrations of up to 26.9 ng/L have been detected in coastal waters near aquaculture sites in Bohai Bay, China, suggesting that these activities significantly contribute to the presence of the antibiotic in the environment [57]. Similarly, a recent study in the Caspian Sea in Iran reported ofloxacin concentrations of up to 235.8 ng/L in surface waters, highlighting the prevalence and potential ecological risk of this compound in the region [64]. The present study detected substantially higher mean and maximum concentrations of ofloxacin, with maximum values of 1293.93 ng/L. These levels markedly exceed those reported in previous studies, suggesting either more intensive use of the antibiotic in the study area or a relationship with the region’s specific characteristics, given its more enclosed environment.

Several studies have documented the presence of ciprofloxacin in aquatic environments. Concentrations of up to 4.6 μg/L have been reported in Australian waters [65]. Even higher levels have been observed in Spain, where maximum concentrations reaching 13.63 μg/L have been detected [66]. Similarly, other authors reported concentrations of this antibiotic as high as 20.31 μg/L on the island of Gran Canaria [7]. In contrast, the results of the present study revealed that ciprofloxacin concentrations were below the limit of quantification (LOQ), indicating a very low or undetectable presence in the sampled area. These differences may be attributed to geographical, methodological, and environmental factors.

Trimethoprim is the most widely used drug of the diaminopyrimidine group. The combination of this antimicrobial together with sulfamethoxazole (sulphamide) to form co-trimoxazole is frequently used in human and veterinary medicine [21]. In this study, the trimethoprim values obtained were higher than those reported in the Baltic Sea (0.5–3.4 ng/L) [67], but lower than those obtained on the Irish coast where it was monitored for one year, reaching maximum concentrations of 870 ng/L [47]. Trimethoprim is considered a pseudopersistent drug in aquatic environments because its residues are consistently present at relatively low concentrations.

### Risk Assessment and Environmental Implications

The widespread presence of pharmaceutical residues in marine environments has been demonstrated by numerous studies conducted worldwide. This situation underscores the importance of assessing the risks posed by exposure to low concentrations of these contaminants in aquatic ecosystems. Evaluating the ecotoxicological risk of pharmaceutical residues, particularly antimicrobial residues, in the aquatic environment is essential.

Toxicity assessments have predominantly focused on invertebrate taxa, including crustaceans, echinoderms, mollusks and polychaete worms, due to their dominance in marine ecosystems in terms of biomass and species diversity [68]. These groups are widely distributed across the globe and exhibit significant ecological and biological variability. Their ability to bioaccumulate contaminants makes them critical vectors for transferring pollutants through marine trophic networks. Their sensitivity to environmental stressors, small body size, short life cycles and ease of laboratory cultivation and field collection, alongside the existence of standardized testing protocols, have also established invertebrates as widely accepted sentinel organisms in marine environmental monitoring programs.

The toxicity of diclofenac to aquatic species is well documented and has been shown to have adverse effects at multiple trophic levels. Exposure to environmentally relevant concentrations of diclofenac induces oxidative stress and impairs the growth, development and behavior of a variety of aquatic organisms. For instance, crustaceans exposed to diclofenac exhibit altered physiological processes, such as abnormal molting and decreased survival rates [69]. Similarly, mollusks show decreased filtration rates and cell damage following exposure [70,71].

Fish species such as *Dicentrarchus labrax* (European sea bass) demonstrate histopathological changes to liver and gill tissues, immunotoxic effects, and reduced reproductive success when exposed to diclofenac at sublethal concentrations [71]. In other fish species such as the common carp (*Cyprinus carpio*), the presence of diclofenac has been shown to induce oxidative stress [72]. Similarly, in rainbow trout (*Oncorhynchus mykiss*), histopathological analyses revealed that exposure to diclofenac causes kidney alterations, including hyaline droplet degeneration of tubular epithelial cells and interstitial nephritis [73]. Furthermore, microalgae, which are key primary producers in aquatic ecosystems, experience inhibition of photosynthetic activity and growth retardation when exposed to diclofenac, which may alter ecosystem productivity [70].

Although the toxicity of fluconazole is less well-studied than that of other contaminants, evidence shows that it has harmful effects on aquatic organisms. It impairs the survival, development and physiological functions of invertebrates, causing antioxidant disruptions and malformations at µg/L levels [63].

In freshwater fish, fluconazole induces immunological changes, histological damage and oxidative stress, as observed in *Clarias gariepinus* [74]. Fluconazole has also been shown to cause cardiovascular toxicity in zebrafish via oxidative stress, apoptosis, and gene disruption [75]. These effects suggest that there are similar risks in marine fish. Its persistence and resistance to biodegradation promote bioaccumulation and chronic exposure, increasing the risk of long-term adverse effects.

Numerous studies [56,62,76] have reported the presence of antibiotics residues in various marine environmental matrices, including remote areas with minimal human activity such as Antarctica [77]. The harmful effects of these antimicrobials, from both acute and chronic exposure, on multiple trophic levels are well documented and affect organisms such as birds, aquatic invertebrates, microalgae, crustaceans and fish.

Experimental exposure to azithromycin, for example, has demonstrated its toxic effects on early developmental stages of European seabass (*Dicentrarchus labrax*), resulting in larval mortality and several lethal malformations in embryos and larvae. Furthermore, azithromycin has been shown to induce oxidative stress, lipid peroxidation damage, and neurotoxicity in juvenile seabass [76].

Table 4 shows the PNEC (ng/L), PNEC-MIC (ng/L) and RQ values for the drugs detected at the Port of Cartagena in Murcia, Spain.

**Figure 5:**
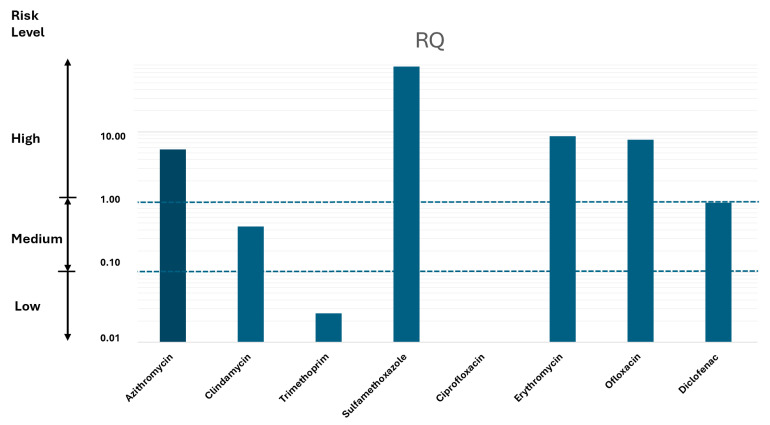
Risk Quotient (RQ) of the investigated drugs.

Figure 5 shows the Risk Quotient (RQ) of the investigated drugs. As can be seen from the data obtained, taking into account the mean concentrations of the drugs tested, four compounds present a high risk (azithromycin, sulfamethoxazole, erythromycin and ofloxacin), one compound presents a moderate risk (clindamycin) and three drugs a low risk (diclofenac, trimethoprim and fluconazole).

The microbial risk quotient (RQMICsub) was evaluated based on the predicted minimum concentrations required to select for antibiotic resistance. The RQMICsub values of azithromycin, sulfamethoxazole and ofloxacin were greater than 1, indicating a high risk of resistance selection at concentrations detected in surface waters. These findings emphasize the importance of paying particular attention to these compounds, given their widespread use and persistence in the environment.

For clindamycin, trimethoprim and erythromycin, RQMICsub values exceeded 0.1, corresponding to a moderate risk. While they did not reach the high-risk threshold, sustained exposure to these sub-inhibitory concentrations could exert selective pressure on microbial communities and contribute to the gradual emergence of resistant strains.

Overall, the results suggest that considering RQMICsub, in addition to traditional ecological risk assessments, may provide a more realistic perspective on the effects of antibiotics in aquatic environments. This approach enables compounds that are not directly toxic to aquatic organisms to be identified as posing a significant threat to the spread of antimicrobial resistance.

Although this pilot study is based on a single sampling campaign and therefore represents a snapshot under specific environmental conditions, the results obtained show the need for further risk studies, with continuous monitoring of seawater, as well as sampling of marine species such as invertebrates, mollusks, fish and harbor birds, in order to characterize in greater depth the extent of the presence of the drugs detected in this environmental setting.

It is noteworthy to mention that for certain target pharmaceuticals, relatively high limits of quantification (LOQs) were obtained due to matrix effects and methodological constraints associated with seawater analysis. Consequently, the absence of detection for these compounds should be interpreted with caution, as their presence at concentrations below the LOQ cannot be excluded. This uncertainty may result in an underestimation of the associated risk quotients (RQs), particularly for compounds with comparatively high LOQs such as ciprofloxacin and ibuprofen. In addition, although the antibiotic profile observed in port waters suggests potential links to human-related sources, such as urban wastewater or ship sewage, a direct comparison with effluents from nearby wastewater treatment plants (WWTPs) was not possible due to the lack of available data. Future research should include increased sampling frequency to better capture temporal variability in pharmaceutical concentrations within port environments. In addition, the implementation of source-tracking approaches, such as the identification of specific discharge points or the use of chemical markers, would help to better characterize emission pathways and distinguish between maritime, urban, and wastewater-related inputs.

## 5. Conclusions

The presence of drugs has been highlighted, mainly the anti-inflammatory diclofenac and the antibiotics azithromycin, erythromycin (both macrolides) and clindamycin, which have been detected in practically all the samples obtained. The concentration values obtained for these are in most cases above the values established for risk analysis and environmental quality values. However, it should be noted that for compounds with relatively high LOQs, non-detections may not necessarily indicate absence in the environment, and associated RQs may therefore be underestimated. To ensure the quality of the water in the port area, even ahead of future requirements, it is necessary to have a continuous monitoring system that allows the presence and concentration of these pollutants to be adequately characterized over time, in order to prevent damage to the environment and biodiversity as well as to enable informed decisions on environmental management in this case in the port area of Cartagena (Murcia, Spain). 

## Figures and Tables

**Table 1:** Limits of detection (LOD), limits of quantification (LOQ) and calibration curve range, expressed in ng/L, for the pharmaceuticals analyzed in this study.

Drugs	LOD	LOQ	Recovery (%)	Calibration Curve Range
Azithromycin	25	50	90	50–5000
Clindamycin	10	25	114	25–2000
Erythromycin	10	25	104	25–2000
Ciprofloxacin	5000	10,000	70	10,000–20,000
Ofloxacin	100	250	82	250–5000
Sulfamethoxazole	10	25	96	25–10,000
Trimethoprim	10	25	94	25–1000
Paracetamol	1000	2500	72	2500–20,000
Diclofenac	10	25	98	25–10,000
Ibuprofen	5000	10,000	70	10,000–20,000
Naproxen	2500	5000	74	5000–20,000
Clotrimazole	25	100	72	100–5000
Fluconazole	100	250	80	250–5000
Venlafaxine	50	250	72	250–5000
Metformin	100	500	70	500–10,000

**Table 2:** Concentration and detection frequencies of the pharmaceuticals detected, expressed in ng/L.

Pharmaceutical	Min	Mean	Max.	DFs (%)
Azithromycin (AZ)	ND	838.28	2206.48	88.89
Clindamycin (CL)	48.42	222.17	608.65	100
Trimethoprim (TMP)	<LOQ	66.76	128.71	77.78
Sulfamethoxazole (SF)	ND	2305.17	8840.61	77.78
Ciprofloxacin (CIP)	ND	<LOQ	<LOQ	33.33
Erythromycin (ER)	64.44	172.95	435.47	100
Ofloxacin (OF)	ND	1231.04	1293.93	33.33
Fluconazole (FL)	ND	428.55	718.31	55.55
Diclofenac (DF)	57.52	942.79	5882.98	100

Min: minimum; Max: maximum, DFs: detection frequency; ND: not detected. Azithromycin (AZ), Clindamycin (CL), Trimethoprim (TMP), Sulfamethoxazole (SF), Ciprofloxacin (CIP), Erythromycin (ER), Ofloxacin (OF), Fluconazole (FL), and Diclofenac (DF).

**Table 3:** Average concentration of the drugs detected at each sampling point (ng/L).

Pharmaceutical	M1.1	M1.2	M1.3	M2.1	M2.2	M2.3	M3.1	M3.2	M3.3
Azithromycin	2206.48	563.13	334.63	924.96	ND	232.20	242.01	822.21	1380.67
Clindamycin	330.71	48.42	608.65	214.27	392.73	52.79	94.30	65.17	192.56
Trimethoprim	128.71	<LOQ	37.52	40.70	60.26	<LOQ	<LOQ	61.12	72.25
Sulfamethoxazole	83.55	ND	<LOQ	223.51	8840.61	<LOQ	ND	73.02	<LOQ
Ciprofloxacin	ND	ND	<LOQ	ND	ND	ND	ND	<LOQ	<LOQ
Erythromycin	435.47	124.13	128.45	305.82	180.51	64.44	144.21	76.74	98.84
Ofloxacin	<LOQ	ND	1293.93	ND	ND	ND	ND	ND	1168.15
Fluconazole	718.31	ND	<LOQ	ND	250,40	ND	ND	316.94	<LOQ
Diclofenac	131.65	57.52	1647.46	196.11	5882.98	92.90	161.23	237.57	77.77

ND: not detected.

**Table 4:** Mean concentrations of the pharmaceuticals detected (ng/L), PNEC (ng/L), PNEC-MIC (ng/L) and RQ values for the drugs detected at the Port of Cartagena in Murcia, Spain.

Pharmaceutical	Mean	PNEC	RQ	PNEC-MIC	(RQ_MICsub_)
Azithromycin [69]	838.28	150	RQ >1	250	RQ >1
Clindamycin [69]	222.17	500	0.1 ≤ RQ < 1	1000	RQ > 0.1
Trimethoprim [70,71]	66.76	2600	0.01 ≤ RQ < 0.1	500	RQ > 0.1
Sulfamethoxazole [70]	2305.17	27	RQ >1	500 *	RQ >1
Erythromycin [70,71]	172.95	20	RQ >1	1000	RQ > 0.1
Ofloxacin [71]	1231.04	160	RQ >1	500	RQ > 1
Fluconazole [72]	428.55	9460	0.01 ≤ RQ < 0.1		
Diclofenac [70]	942.79	970	0.01 ≤ RQ < 0.1		

* Pnec-MIC for Trimethoprim-Sulfametoxazol. RQ > 1 indicates high risk, 0.1 ≤ RQ < 1 indicates moderate risk, 0.01 ≤ RQ < 0.1 indicates low risk, and RQ ≤ 0.01 indicates insignificant risk.

## Data Availability

The data supporting the conclusions of this article will be made available by the corresponding author upon reasonable request.

## Supplementary Materials

The following supporting information can be downloaded at: https://www.mdpi.com/article/10.3390/toxics14030217/s1, Figure S1: Representative MRM chromatograms of standards sample; Figure S2: Representative MRM chromatograms of spiked sample; Figure S3: Representative MRM chromatograms of an environmental sample; Table S1: Physical and chemical parameters determined during the month of February 2025 at different sampling points; Table S2: Mass spectrometer parameters for the determination of target analytes in negative mode; Table S3: Mass spectrometer parameters for the determination of target analytes in positive mode; Table S4: PNEC value used, the test organism, the toxicity endpoint (LC50), and the assessment factor applied (AF) for each detected drug.
