# Supplementary material for: Occurrence of Pharmaceuticals in the Seawater Samples of the Port of Cartagena (Murcia, Spain): A Pilot Study
Source: Toxics. 2026 Mar 3;14(3):217. doi: 10.3390/toxics14030217 (PMC13030690; doi:10.3390/toxics14030217)

Figure S1: Representative MRM chromatograms of standards sample

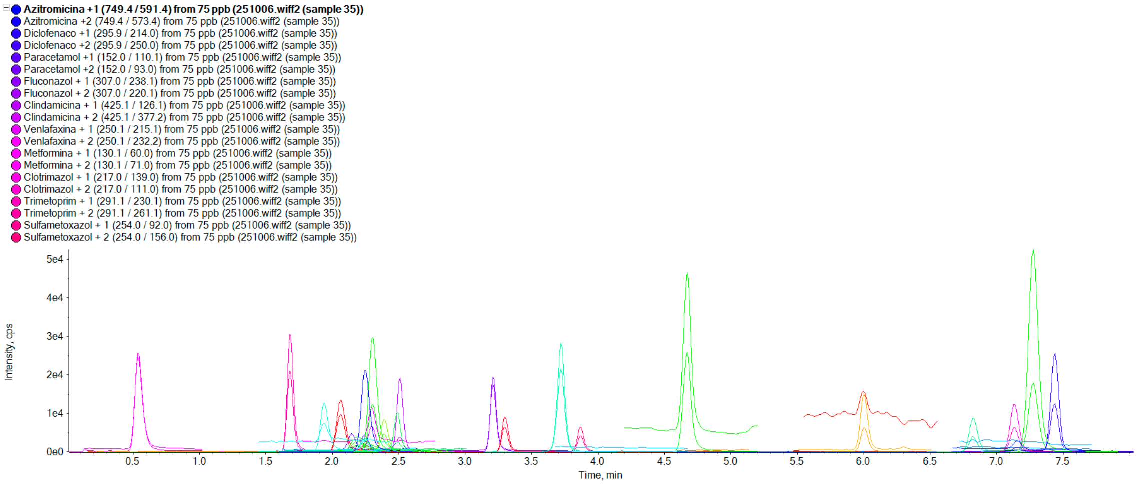

Figure S2: Representative MRM chromatograms of spiked sample

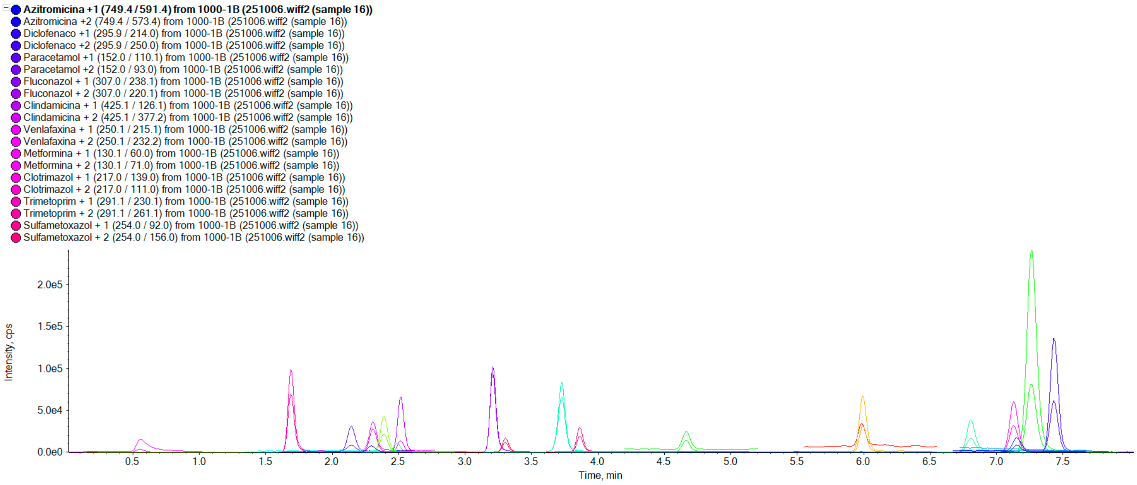

Figure S3: Representative MRM chromatograms of an environmental sample

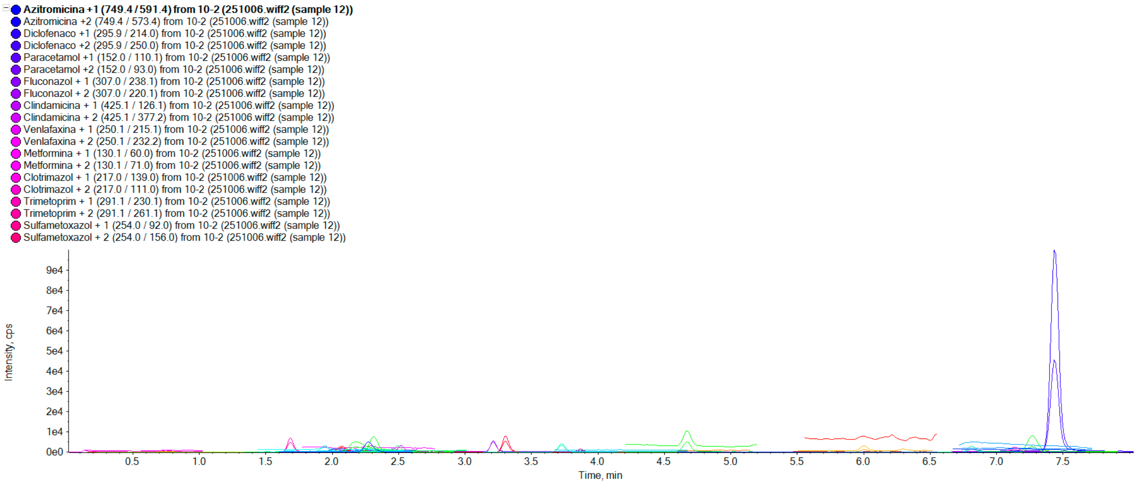

Supplement: Supplementary file 1 [file toxics-14-00217-s001.zip › Figures SX.pdf]
