# Supplementary material for: Occurrence of Pharmaceuticals in the Seawater Samples of the Port of Cartagena (Murcia, Spain): A Pilot Study
Source: Toxics. 2026 Mar 3;14(3):217. doi: 10.3390/toxics14030217 (PMC13030690; doi:10.3390/toxics14030217)
Supplement: Supplementary file 1 [file toxics-14-00217-s001.zip › Table S1.pdf]

Table S1. Physical and chemical parameters determined during the month of February 2025 at different sampling points.

SAMPLING POINT	T (°C)	SpCond (mS/cm)	Sal psu	ODO % sat	ODO mg/L	pH	ORP mV	Chlorophyll (µg/L)
M 1.1	17.097	53.5967	35.45	96.7	7.53	8.26	200.1	0.76
M 1.2	16.873	53.6204	35.46	99.2	7.76	8.27	200.9	0.88
M 1.3	16.84	53.1042	35.08	103.3	8.11	8.27	202.5	1.21
M 2.1	16.759	53.6622	35.49	104.5	8.19	8.34	196.8	0.68
M 2.2	16.923	53.6648	35.5	105.5	8.25	8.34	198.7	0.47
M 2.3	17.094	53.7102	35.53	102.4	7.97	8.31	193.4	0.26
M 3.1	16.953	53.6333	35.47	103.8	8.1	8.33	199.8	0.52
M 3.2	16.968	53.7557	35.57	102.7	8.01	8.31	195.6	0.29
M 3.3	16.956	53.6417	35.48	103.4	8.08	8.3	199.4	0.77

Temperature (T), electrical conductivity (SpCond), dissolved oxygen (ODO), salinity (Sal), chlorophyll concentration (Chlorophyll) and oxidation-reduction potential (ORP).
