# Supplementary material for: Occurrence of Pharmaceuticals in the Seawater Samples of the Port of Cartagena (Murcia, Spain): A Pilot Study
Source: Toxics. 2026 Mar 3;14(3):217. doi: 10.3390/toxics14030217 (PMC13030690; doi:10.3390/toxics14030217)
Supplement: Supplementary file 1 [file toxics-14-00217-s001.zip › Table S2.pdf]

Table S2. Mass spectrometer parameters for the determination of target analytes in negative mode.

Drug	*Retention time (min)*	*Masa Q1 (Da)*	*Masa Q3 (Da)*	*Dwell time (ms)*	*Decomposition potential (V)*	*Collision energy (V)*
Ibuprofen	7.22	205.068	161	81.1868686868687	-30	-12
Ibuprofen	7.22	205.068	159.2	81.1868686868687	-30	-11
Naproxen	6.05	229.015	168.94	225	-25	-40
Naproxen	6.05	229.015	140.946	225	-25	-63
