# Supplementary material for: Occurrence of Pharmaceuticals in the Seawater Samples of the Port of Cartagena (Murcia, Spain): A Pilot Study
Source: Toxics. 2026 Mar 3;14(3):217. doi: 10.3390/toxics14030217 (PMC13030690; doi:10.3390/toxics14030217)
Supplement: Supplementary file 1 [file toxics-14-00217-s001.zip › Table S3.pdf]

Table S3. Mass spectrometer parameters for the determination of target analytes in positive mode.

Drug	*Retention time (min)*	*Masa Q1 (Da)*	*Masa Q3 (Da)*	*Dwell time (ms)*	*Decomposition potential (V)*	*Collision energy (V)*
Azithromycin	2.25	749.3646	591.3832	34.5040761707428	30	41
Azithromycin	2.25	749.3646	573.3799	34.5040761707428	30	49
Azithromycin	2.25	749.3646	83.0197	34.5040761707428	30	108
Diclofenac	7.53	295.9398	214.0311	117.80303030303	20	49
Diclofenac	7.53	295.9398	250.0201	117.80303030303	20	18
Paracetamol	2.14	152.035	110.1	33.3315122377623	70	23
Paracetamol	2.14	152.035	93	33.3315122377623	70	29
Fluconazole	3.21	307.0398	238.0638	113.862781954887	55	23
Fluconazole	3.21	307.0398	220.0898	113.862781954887	55	24
Clindamycin	2.44	425.1207	126.1391	49.3894802635404	120	33
Clindamycin	2.44	425.1207	377.164	49.3894802635404	120	27
Venlafaxine	2.2	250.0895	232.1558	33.4624095040762	30	12
Venlafaxine	2.2	250.0895	215.1481	33.4624095040762	30	17
Metformin	0.52	130.109	60.0364	136.458333333333	35	17
Metformin	0.52	130.109	71.022	136.458333333333	35	28
Clotrimazole	7.16	216.986	139.009	88.510101010101	75	23
Clotrimazole	7.16	216.986	111.011	88.510101010101	75	45
Trimethoprim A	0.75	291.056	230.144	118.229166666667	100	32
Trimethoprim A	0.75	291.056	261.081	118.229166666667	100	34
Trimethoprim B	1.6	291.056	230.144	99.6343021014073	100	32
Trimethoprim B	1.6	291.056	261.081	99.6343021014073	100	34
Sulfamethoxazole	3.88	254.005	155.998	126.909722222222	60	23
Sulfamethoxazole+	3.88	254.005	92.012	126.909722222222	60	36
Ciprofloxacin	2.15	332.098	314.095	33.2410218923377	115	30
Ciprofloxacin	2.15	332.098	231.079	33.2410218923377	115	52
Erythromycin	3.62	748.335	590.34	97.3958333333334	80	28

Erythromycin	3.62	748.335	158.125	97.3958333333334	80	37
Ofloxacin	1.98	362.056	318.127	44.931891025641	35	27
Ofloxacin	1.98	362.056	261.098	44.931891025641	35	38
