# Supplementary material for: Occurrence of Pharmaceuticals in the Seawater Samples of the Port of Cartagena (Murcia, Spain): A Pilot Study
Source: Toxics. 2026 Mar 3;14(3):217. doi: 10.3390/toxics14030217 (PMC13030690; doi:10.3390/toxics14030217)
Supplement: Supplementary file 1 [file toxics-14-00217-s001.zip › Table S4.pdf]

Table S4. PNEC value used, the test organism, the toxicity endpoint (LC50), and the assessment factor applied (AF) for each detected drug.

Compounds	Species assayed	Test (endpoint)	Toxicity (mg/L)	AF	PNEC (μg/L)	References
**Diclofenac**	Fish	EC50 ECOSAR	532	1000	**9.7**	[31]
	Daphnia	EC50 ECOSAR	5057	1000		[31]
	Algae	EC50 ECOSAR	2911	1000		[31]
	Daphnia	EC50 (48 h)	22.43	1000		[32]
	C. dubia	EC50 (48 h)	22.7	1000		[32]
	Daphnia	EC50 (48 h-mortality)	22.4	1000		[33]
	Algae	EC50 (96 h-growth)	16.3	1000		[33]
	Bacteria	EC50 (30 min-luminescence)	11.4	1000		[33]
	Bacteria	EC50 (15 min-inhibition)	9.7	1000		[34]
	Microtox	EC50 (30 min)	11.45	1000		[32]
	Algae	EC50 (96 h-growth)	14.5	1000		[33]
	Invertebrates	EC50	90	1000		[35]
	Algae	EC50-inhibition	72	1000		[36]
	Daphnia	EC50-immobilisation	68	1000		[36]
**Azithromycin**	-	-	0.15	1000	0.15	[37]
**Clindamycin**	-	-	0.5	1000	0.5	[37]

For azithromycin and clindamycin, the PNEC was derived from a single toxicity value reported by Kümmerer and Henninger (2003), due to the lack of multispecies EC50 data.

Compounds	Species assayed	Test (endpoint)	Toxicity (mg/L)	AF	PNEC (μg/L)	References
**Sulfamethoxazole**	Fish	EC50 ECOSAR	890	1000	0.027	[31]
	Daphnia	EC50 ECOSAR	4.5	1000		[31]
	Algae	EC50 ECOSAR	51	1000		[31]
	Fish	EC50 (96 h)	563	1000		[38]
	Daphnia	EC50 (48 h-mortality)	>100	1000		[33]
	Bacteria	EC50 (15 min)	78.1	1000		[38]
	Algae	EC50 (96 h-growth)	0.15	1000		[33]
	Algae	EC50 (96 h-growth)	0.027	1000		[33]
**Trimethoprim**	Fish	EC50 ECOSAR	795	1000	2.6	[31]
	Daphnia	EC50 ECOSAR	4.8	1000		[31]
	Algae	EC50 ECOSAR	2.6	1000		[31]
	Bacteria	EC50 (15 min)	177	1000		[38]
	Daphnia	EC50 (96 h-immobility)	121	1000		[38]
	Fish	EC50 (48 h)	>100	1000		[38]
	Invertebrates	EC50	110	1000		[35]
	Algae	EC50	90	1000		[35]
	Fish	EC50	100	1000		[35]

Compounds	Species assayed	Test (endpoint)	Toxicity (mg/L)	AF	PNEC (μg/L)	References
**Erythromycin**	Fish	EC50 ECOSAR	61	1000	0.02	[31]
	Daphnia	EC50 ECOSAR	7.8	1000		[31]
	Algae	EC50 ECOSAR	4.3	1000		[31]
	Invertebrates	EC50	15	1000		[35]
	Algae	EC50	0.02	1000		[35]
	Fish	EC50	900	1000		[35]
**Fluconazole**	L. minor	EC10 (growth, CI)	0.473	50	9.46	[39]
	R. subcapitata	EC10 (growth, CI)	26.8	50		[39]
	D. magna	EC10 (CI)	20.0	50		[39]
**Ofloxacin**	Algae	EC50 (96 h-growth)	0.016	1000	0.016	[33]
	Invertebrates	EC50	30	1000		[35]
	Algae	EC50	1.5	1000		[35]
	Fish	EC50	10	1000		[35]

Predicted No-Effect Concentration (PNEC) values were extracted from the review by **Verlicchi et al.** and used to evaluate the environmental risk of pharmaceutical compounds through standard risk quotient calculations. Additional scientific literature, including the attached bibliography, was consulted to support and validate the risk assessment framework.
